# Sodium Content of Foods Sold in the Spanish Market. Results from the BADALI Project

**DOI:** 10.3390/nu13103410

**Published:** 2021-09-27

**Authors:** Marta Beltrá, Fernando Borrás, Ana B. Ropero

**Affiliations:** 1 Institute of Bioengineering, Miguel Hernández University, 03202 Elche, Spain; beltra@umh.es; 2 Department of Statistics, Mathematics and Informatics, Miguel Hernández University, 03202 Elche, Spain; f.borras@umh.es

**Keywords:** salt content, nutrient composition, nutritional claims, nutrient profile/profiling models, changes in sodium content, food database, public health

## Abstract

High sodium/salt intake is a risk factor for Non-Communicable Diseases (NCDs). Excess sodium intake has been associated with high coronary heart disease, stroke and high blood pressure. The sodium daily intake is above the recommendations in the world as well as in Spain. Reducing salt content in processed foods and ready meals is one of the main strategies for reducing sodium intake. The aim of the present work is to characterise the presence of sodium in foods sold in the Spanish market. We also study a possible shift in sodium content in products over the last few years. For this purpose, 3897 products included in the BADALI food database were analysed, classified into 16 groups (G). We found that 93.3% of all foods displayed the sodium/salt content in the nutrition declaration. Meat—processed and derivatives (G8) had the highest mean and median values for sodium content, followed by snacks (G15) and sauces (G14). Only 12.7% of foods were sodium-free (≤5 mg/100 g or 100 mL), 32.4% had very low sodium (≤40 mg/100 g or 100 mL) and 48.2% were low in sodium (≤120 mg/100 g or 100 mL). On the contrary, 47.2% were high in sodium according to the Pan American Health Organisation Nutrient Profile Model (PAHO-NPM), while there were 31.9% according to the Chile-NPM. The agreement between the two NPMs was considered ‘substantial’ (κ = 0.67). When sodium content was compared over the years, no decrease was observed. This analysis was performed in the entire food population, by food group and in matched products. Therefore, more effort should be made by all parties involved in order to decrease the sodium/salt intake in the population.

## 1. Introduction

Reduction in salt intake was considered by the World Health Organization (WHO) in 2018 as one of the best investments to reduce Non-Communicable Diseases (NCDs) [1]. NCDs are the leading cause of death in the world. It is estimated that they are responsible for 41 million deaths in the world each year, which represents 71% of all deaths [2]. Cardiovascular diseases (CVD) and cancer account for most of those deaths [2]. In Spain, the Global Burden Disease (GBD) 2019 and data from WHO showed that 9 out of the 10 main causes of death are NCDs [3,4,5].

Raised blood pressure is the leading metabolic risk factor in the world contributing to NCDs [2]. High sodium/salt intake has been associated with high blood pressure and is a risk factor for NCDs [6,7]. In addition, excess sodium intake has been related to coronary heart disease and stroke [6].

According to the World Cancer Research Foundation (WCRF), there is strong evidence that consuming foods preserved by salting is a cause of stomach cancer [8]. High dietary salt has also been shown to adversely affect the vasculature, heart, kidneys, skin, brain and bone [9].

As a consequence of all the evidence, WHO stablished the maximum recommended sodium intake for adults in 2 g/d in 2012 [10]. According to the GBD, the global mean intake of sodium was 3.95 g/d in 2010 [11]. Salt/sodium intake in the Spanish population is also higher than recommended. The last estimation performed by 24 h urinary sodium excretion was of 9.8 g salt/d, with 88.2% of the subjects with intakes above 5 g/d [12]. More recent data from the ANIBES study, following a three-day food records, also showed an excess of sodium intake in Spain [13].

Randomized trials demonstrate that salt reduction lowers blood pressure in normotensive, as well as in hypertensive individuals additively to antihypertensive treatments [14]. Studies have shown that decreasing salt intake is associated with reduced risk of CVD, all-cause mortality, kidney disease, stomach cancer and osteoporosis [14]. A recent study estimated the impact of the salt reduction program in England. Salt intake decreased from 2000 to 2018 [15]. Authors calculated that maintaining the salt intake at 2018 levels would reduce considerable the cases of premature ischemic heart disease and strokes [15]. This would generate more than half million of extra quality-adjusted life-years and £1640 million health care cost savings for the adult population in England [15].

Most sodium intake in Europe and Northern American countries comes from salt added in manufactured foods (around 75% of the total intake) [16]. Therefore, reducing salt content in processed foods and ready meals is one of the main strategies for decreasing sodium intake in the population [17]. Salt reducing programs have been ongoing for some years in countries such as UK [18], Canada [19], Argentina [20], Brazil [21,22], Italy [23] and South Africa [24]. Recently, WHO released global sodium benchmarks depending on the food category [25]. Maximum sodium values were set in those programs for food groups such as bread, processed meat and fish, canned vegetables and legumes, snacks, breakfast cereals, sauces, among others.

In 2018, the Spanish Agency for Food Safety and Nutrition (AESAN), along with food professionals, released the Plan for the Improvement of the Composition of Food, Beverages and Other Measures 2020 for the period 2017–20 [26]. Reducing added sugar, salt and trans fatty acids content in foods were the main targets [26]. Snacks, processed meat, sauces, vegetable purees, ready to eat and precooked foods were the groups included in the plan for a 5–16% reduction in sodium content [26]. Joining the plan was voluntary.

In the last few years, the sodium content of foods has been studied over time. Results are diverse and depend on the food category and the country of study [27,28,29,30,31,32,33,34]. In Spain, a government report in 2015 showed a decrease in sodium content in some food categories in 2012 compared to 2009 [35]. No scientific publication has been released so far with the results of the Plan 2020.

The aim of the present work is to characterize the presence of sodium in foods sold in the Spanish market in recent years and to analyse a possible reduction over time. This study will focus on food groups.

## 2. Materials and Methods

### 2.1. BADALI Database of Food Products Available in the Spanish Market

The data used in this work come from the BADALI database project [36,37]. Details about the food and brand selection process can be found in Ropero et al., 2020 [38]. In short, the information used in this study was obtained from the manufacturers’ web pages, including the nutrient composition and ingredients. Serving size for precooked and ready-to-eat foods was also obtained from online supermarkets (June 2021).

Nutrient composition of foods was extracted by the researchers and inconsistent information was not used for further analysis. For the purpose of this study and in order to reduce heterogeneity, foods were classified following similarities in the main ingredients, use and/or sodium content (Table S1). Fresh foods were poorly represented in the database, the main exception being fish and seafood (included in G10). For the calculation of the percentage of sodium daily intake, 2 g sodium/d was applied [10].

Two versions of the database were utilised for the present study. The oldest version was used only for the baseline sodium content in the comparative study. It included foods collected from June 2014 to April 2019. The newest version of the database was used for all the analyses throughout this work. It is comprised of all the foods in the previous version, except for those collected before January 2017, which were removed. In addition, the information on some foods was updated, and new information was added (from October 2020 to May 2021).

### 2.2. Classification of Products According to Their Sodium Content

For the classification of foods as “low in sodium”, “very low in sodium” or “sodium-free”, the criteria established in the Regulation (EC) No 1924/2006 and the Codex Alimentarius for the respective nutrition claims were used (Table 1) [39,40].

Two criteria were applied to classify foods as high in sodium (Table 1). On one hand, the Pan American Health Organization Nutrient Profile Model (PAHO-NPM) [41] and, on the other hand, the Chilean warning label system established by the Minister for Health (Chile-NPM) [42]. These NPMs have been previously used to determine the “healthiness” of foods, based on their content of several nutrients [43,44,45]. In addition, their criteria for sodium/salt was also used to classify foods as high in sodium, independently of the presence of other nutrients [46].

According to PAHO, the food and beverage products that should be evaluated with their NPM are limited to processed and ultra-processed products, which typically contain elevated amounts of sodium, free sugars, saturated fat, total fat and trans-fatty acids added by the manufacturer. There is no reason to apply the PAHO-NPM to unprocessed or minimally processed foods [41]. As for the Chile-NPM, the labelling of products as high in sodium, energy, total sugar or saturated fats is not compulsory for foods without added sugar, honey, sodium or saturated fats [42]. In spite of these restrictions, we decided to apply both NPMs to all foods in the database as this is a research project and not a public health initiative.

### 2.3. Comparison of Sodium Content over the Years

The two versions of the database described in Section 2.1 were used. Since some items were present in both versions, duplicates were removed prior to the analysis. For the matching comparison, identical products were chosen in different years (2–6 years gap). Small differences were permitted, given that the product didn’t undergo major changes.

### 2.4. Statistics

The Kruskal-Wallis test is useful as a general nonparametric test for comparing more than two independent samples. It can be used to test whether such samples come from the same distribution. This test is a powerful alternative to the one-way analysis of variance. Nonparametric ANOVA has no assumption of normality of random error but the independence of random error is required. If the Kruskal-Wallis statistic is significant, the nonparametric multiple comparison tests are useful methods for further analysis.

Pairwise agreement between both NPMs in the proportions of foods classified as “high in sodium” was assessed across all foods using the κ statistics, as follows: 0.01–0.20 ‘slight’; 0.21–0.40 ‘fair’; 0.41–0.60 ‘moderate’; 0.61–0.80 ‘substantial’; 0.81–0.99 ‘near perfect’. When agreement is high, the κ statistics either cannot be calculated or provides inconsistent values. Therefore, for some groups the agreement was assessed by using the disagreement probability (0 to 1). When this parameter was >0.1, it was considered ‘substantial’; <0.1 ‘near perfect’ and 0 ‘perfect’. The statistical analysis of the application data in this work was performed with Microsoft Excel and Google Colab with Jupyter Notebooks, libraries scikit-learn 0.22.2.post1, Pandas v0.25.3, and Matplotlib Python v3.2.0. The significance level was set as *p* < 0.05 in all statistical analyses.

## 3. Results

### 3.1. Data Description and General Overview

A total of 3897 products were collected from 2017 to 2021, belonging to 169 well identified brands and classified into groups as in Table S1. As shown in Table 2, the most abundant food groups were dairies and substitutes (G5), sweets (G16) and the one-type of ingredient group (G10). The least abundant was that of fats (G6).

Of the total population, 93.3% displayed the sodium or salt content (Table 2), while 263 foods did not. Precooked and ready-to-eat food (G13) displayed the sodium/salt content in all the items, while the one-type of ingredient group (G10) only in 70.7% of the cases. In 18 foods (0.5%) an error was detected. Therefore, a total of 3616 foods were subsequently used for further analysis.

The sodium content fell below 2500 mg in all items, except for 10 foods (Figure S1). For these, the values were in the range of 3480–5200 mg sodium/100 g and eight of them were canned anchovies (Figure S1—insert). Median sodium content was highest for meat—processed and derivatives (G8), followed by snacks (G15) and sauces (G14) (Table 3). Five groups had median sodium values below 50 mg/100 g: dairies and substitutes (G5), non-alcoholic drinks (G9), one-type of ingredient (G10), pasta (G12) and sweets (G16) (Table 3). These groups displayed a narrow dispersion of values (Figure S1). In fact, foods in G10 (one type of ingredient) and G12 (pasta) had no added salt. Two powdered milk in G5 (dairies and substitutes) and four tomato juices in G9 (non-alcoholic drinks) (with added salt) were exceptions with unusually high sodium content (Figure S1).

Some other interesting results could be observed in Table 3. The sodium content of some particular food types was also calculated and shown in Table S2. In spite of their sweet taste, cereal sweet derivatives (G3) had considerable amounts of sodium, corresponding to added salt. Breakfast cereals and bars had the lowest values within this group (Table S2). Cheese (G4) could be classified into two types: fresh/soft cheese had lower sodium content than mature (Table S2). Dairies had slightly higher sodium content than substitutes and emulsion-based sauces had lower values than the rest of the foods in this group (Table S2). The sodium content of cereal-based snacks was higher than for nuts and vegetables (Table S2).

For precooked and ready-to-eat food (G13), the sodium content per serving recommended by the manufacturer could be calculated for 141 products (Table S3). The median sodium content per serving was 507.5 mg. The percentage of the daily reference intake (RI) was determined for all of them and the median was 25.4% (Table S3).

### 3.2. Food Classification According to Their Sodium Content

**Figure 1:**
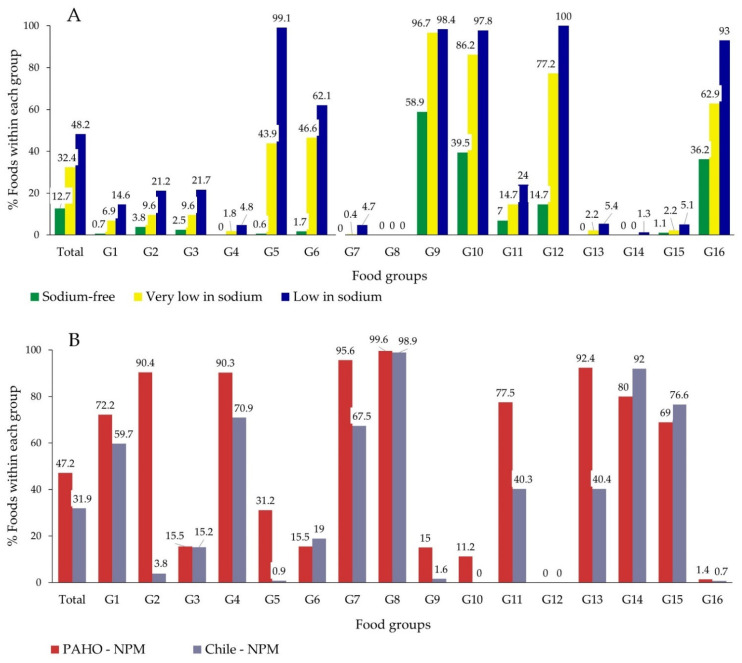
Foods in conformity with the nutrition claims regulated by the European Regulation No 1924/2006 [39] and the Codex Alimentarius [40] (**A**) or exceeding the NPMs thresholds (**B**) for sodium, by group.

As shown in Figure 1A, only 12.7% of foods complied with the conditions for the nutrient claim sodium-free and most of them belonged to G9 (non-alcoholic drinks), G10 (one type of ingredient) and G16 (sweets) (Table S4). Around one third of foods could be categorized as very low in sodium (32.4%), while 48.2% of foods were low in sodium (Figure 1A).

Five groups had more than 90% of their items classified as low in sodium (G5, G9, G10, G12 and G16) (Figure 1A). Not surprisingly, none of the foods in G8 (meat) qualified for any nutrient claim on sodium. Only one could be classified as low in sodium in G14 (snacks), while less than 5% in G4 (cheese) and G7 (fish/seafood). G9 (non-alcoholic drinks) was the only group with a considerable proportion of sodium-free foods (58.9%), while for G10 (one type of ingredient) and G16 (sweets) values were below 40% (Figure 1A).

When the Nutrient Profile Models (NPMs) were applied, opposite results were obtained (Figure 1B). Near half of all foods were considered high in sodium according to the PAHO-NPM (47.2%), while around one third with the Chile-NPM (31.9%). More than 10% of all foods classified as high in sodium by both NPMs belonged to G7 (fish/seafood), G8 (meat) and G15 (snacks) each (Table S4). Only 1 and 3 foods out of 280 in the meat group (G8) was not considered high in sodium according to the PAHO-NPM and Chile-NPM respectively (Figure 1B). G4 (cheese), G7 (fish) and G14 (sauces) had also a very large proportion of foods high in sodium (Figure 1B). On the contrary, no food exceeded the maximum values in G12 (pasta), while only a few in G16 (sweets) (3 and 6 foods according to the PAHO-NPM and Chile-NPM respectively) (Figure 1B, Table S4).

It is interesting to note that the results obtained by applying both NPMs strongly differed in some groups (Figure 1B, Table S4). The κ statistics and the disagreement probability were used to compare both NPMs on foods (Table 4). The agreement was considered “substantial” for the total food database. However, important discrepancies were obtained for some food groups. On one hand, both NPMs were in accord for the G12 and had a “near perfect” agreement for G3, G6, G8 and G15. On the other hand, it was only “slight” for G2, G5, G7, G9, G13 and G14.

### 3.3. Changes in Sodium Content over the Years

When sodium content was compared over the years, some differences were observed. Foods from 2020–21 had the highest sodium content (Table 5). This may be due to different kinds of foods collected over the years. In order to minimise this confounding factor, a comparison was performed by food group. Only those with at least 30 items from three different brands were considered for this analysis (G6, G7 and G13 did not meet this requirement). Groups with no added salt (G9, G10, G12) or high heterogeneity (G11) were also discarded.

As seen in Table 5, G4 (cheese), G5 (dairies and substitutes), G8 (meat) and G14 (sauces) showed statistically significant increases in sodium content in 2020–21 compared to 2017–19. No tendency to decrease sodium content was observed for any group analysed.

Further analysis was performed by comparing the same products over the years (matching products). A total of 219 foods could be studied, from 29 different brands and belonging to 12 groups. No differences were observed in sodium content (mean sodium differences = −7.1 mg/100 g; median sodium differences = 0 mg/100 g; 25th and 75th were also 0 mg/100 g).

## 4. Discussion

The present work analyses 3897 foods sold in the Spanish market from 2017 to 2021. Sodium content depends much on the food group. A low proportion of foods were sodium-free and almost half of foods were low in sodium. A high proportion of foods were considered high in sodium according to the two NPMs used. Both NPMs greatly disagreed in some food groups. No decrease in sodium content was observed over the years.

### 4.1. Sodium/Salt Content in Foods

To our knowledge, this is the first paper published in a scientific journal studying the sodium/salt content of diverse foods sold in the Spanish market. However, a previous report by the Spanish Government in 2012 showed similar results for meat, sauces, bread, cereal sweet derivatives, precooked and ready-to-eat food [35]. Our results produced higher sodium content for snacks, canned fish/seafood and cheese, while lower for canned vegetables [35].

A work on sodium content in bread in Spain was previously published in 2018 [47]. They obtained a much higher mean in bread purchased in bakeries (see Table S5) [47]. One important reason for the discrepancy may be that no bakery bread is included in the present study, but industrial bread and other similar products. In addition, the Spanish Government issued a regulation in 2019 to limit the sodium content in bread [48].

As it can be observed in Table S5, the present results are in line with preceding works [49,50,51,52,53,54]. The sodium/salt content of foods has been studied in the last five years in a number of countries (Table S5). Meat is the food group with the highest mean/median sodium content in all the countries (except for sauces in some of them) (Table S5). The values for most food groups do not vary greatly among studies (including the present one), except for sauces. Still, the discrepancies may be due to the diverse definition of the food categories (see comments in Table S5). In fact, unlike the present work, most of the publications do not describe the food groups.

### 4.2. Classification of Foods According to Their Sodium/Salt Content

To our knowledge, this is the first paper using the entire set of nutrient claims for salt/sodium defined by the European Commission (EC) and the Codex Alimentarius to classify foods [39,40]. However, the definition for low in sodium was previously used in a Brazilian study and it rendered 7% of the 1416 foods analysed [55]. With the same threshold, we found higher numbers (48.2%).

Our results show a great proportion of foods as high in sodium. By applying the same NPMs, a study in Honduras with 1009 foods obtained higher values: 55.8% according to PAHO-NPM (47.2% in the present work) and 68.6% when using the Chile-NPM (31.9% here) [45]. The differences could be due to the type of foods used in both studies. The work in Honduras only analysed processed and ultra-processed foods as defined by the NOVA classification [45]. However, in the present work, the NPMs were applied to all foods regardless of their level of processing.

Applying both NPMs resulted in some important differences in the present work. More foods were classified as high in sodium with the PAHO-NPM than with the Chile-NPM. The study in Honduras obtained opposite results, which may be due to the same reasons explained in the previous paragraph [45]. In addition, individual groups presented even greater discrepancies, which was also the case in the work in Honduras. This may be because, on one hand, the criteria for the PAHO-NPM is based on sodium per kcal regardless of the type of food [41]. On the other hand, the thresholds for Chile-NPM only consider the sodium content and they differ for solids and liquids [42]. Divergences are not exclusive of these two NPMs [44,45].

Recently, WHO released global sodium benchmarks for more than 50 food subcategories. Table S2 shows the thresholds for some of the food types analysed in this work. Except for breakfast cereals and salads, the median values of the rest of food types surpassed the benchmarks set by WHO [25].

### 4.3. Reduction in Sodium/Salt Content

In the present work, no reduction of sodium content was obtained over time either in the total food sample or in any of the nine food groups analysed. Previous papers have shown diverse outcomes. A study in Canada compared more than 6000 foods/year in 2010 vs. 2013 [32]. The authors found a significant reduction of sodium content only in 16.2% of foods categories, while no changes were observed in 81.9% of them [32]. An analysis performed in Costa Rica on more than 1000 foods/year showed decreased mean sodium content in 3 out of 18 food categories, cakes being one of them [27]. Similarly, an Indian work with 1407 products, only found a reduction in ready meals and canned vegetables, while sodium increased in 5 out of 29 food categories [30]. A paper studying salt content in sauces in the UK showed a significant reduction in median salt content in eight out of seventeen sauce categories [31].

A comparison of 219 matched products did not show a reduction in sodium content in the present paper (2017–19 vs. 2020–21). A report by the Spanish Government compared matched or similar products between 2009 and 2012 [35]. They found that sodium content decreases in breakfast cereals, soups, canned fish/seafood and industrial bread. On the contrary, sodium values increased in processed meat and sauces, which is in line with our results [35].

No changes in sodium content over time were found in a study in New Zealand comparing 182 products in 2003 vs. 2013 [33]. The same results were obtained in a food sample in Slovenia (98 foods, 2011 vs. 2015) [29]. However, an overall reduction of 23% in sodium content was obtained in a sample of 130 foods in the Australian market in 2013 vs. 1980 [34]. The comparison of 2979 matched products in the USA showed a statistically significant reduction of sodium content in 13 out of the 14 food groups analysed (2009 vs. 2015) [28].

As it seems, there is not a consensual reduction in sodium/salt content in foods in recent years. Neither the Spanish Plan for the Improvement of the Composition of Food, Beverages and Other Measures 2020 [26] has produced an effective decrease in sodium content in snacks, processed meat and sauces, according to our results. It is feasible that changes may only be detected in large samples, due to variability and bias in the collected information. However, the most probable reason is that the sodium content in foods has not really decreased over the last years.

### 4.4. Sodium/Salt in the Diet

As mentioned in the Introduction, daily sodium intake in the world and in Spain exceeds the recommendations [11,12,13]. According to the ANIBES study, processed meat and bread are the main dietary sources of sodium intake in the Spanish population [13]. Our results show that processed meat and derivatives was the food group with the highest sodium content values and that it increased over the years. In 2012, a voluntary agreement between the Spanish Agency for Food Safety and Nutrition (AESAN), the Spanish Confederation of Meat Retailers (CEDECARNE) and the Association of Manufacturer and Retailers of Food Additives and Supplements (AFCA) was signed to decrease the sodium content of these foods [56]. Whether that actually happened or not at that time, our results show that there are still reasons to be concerned about the high sodium content of processed meat.

Regarding bread, the Spanish Government released a decree in 2019 establishing a maximum sodium content for bread of 520 or 660 mg sodium/100 g, depending on the analytical method used [48]. Our data show a lower sodium content even before the decree was enforced (years 2017–19). We should mention that bread elaborated in bakeries was not included in our database while other bread-like foods were.

### 4.5. Strengths and Limitations

The present work has some important strengths:

- This is the first paper published in a scientific journal studying the sodium/salt content of diverse foods sold in the Spanish market;
- Foods from all groups were analysed, which provided an overview of the Spanish market;
- More than 3800 foods were analysed and the number of foods per group was significant;
- Most foods included in the database were processed, which usually have added salt.
- Data was collected following criteria completely unrelated to the aim of this study or the targeted population and, as a consequence, our results lack any bias on food choice;
- The comparative analysis of sodium content was performed at different levels in order to minimize heterogeneity in foods included in the database in different years.

The limitations are also to be mentioned:

- Data collected were reliant on the accuracy of the information provided on the manufacturer’s webpage;
- Selection of brands did not follow criteria based on customer’s purchase or the most popular products;
- The 3897 foods analysed may not be representative of the Spanish market due to the huge amount of foods available;
- Many of the products displayed 0 g salt/sodium, which could be wrongly rounded. The EC published a guidance document with rounding instructions, but it is not compulsory [57].
- The number of products for the matched comparative study was low, although in line with most of previous studies.

## 5. Conclusions

The results of our study reveal that sodium content in foods in the Spanish market is very high. Much work is still ahead of us in order to achieve the WHO challenge of reducing the sodium intake of the population by 30% by 2025. The Spanish Plan for the Improvement of the Composition of Food, Beverages and Other Measures 2020 [26] has not produced an effective decrease in sodium content in some of the food groups targeted. The voluntary nature of the agreement with food professionals is clearly insufficient to produce positive results. Unless mandatory regulations were issued, the effectiveness of such programs will be very limited. A firm compromise of all parties involved, governments, industry and consumers, is required for demanding, enforcing and monitoring a truly effective program.

## Figures and Tables

**Table 1:** Criteria used to classify foods according to their sodium content.

Criteria	Claim	Threshold-Sodium
Regulation (EC) No 1924/2006 and Codex Alimentarius [39,40]	Sodium-free	≤5 mg/100 g or 100 mL
	Very low in sodium	≤40 mg/100 g or 100 mL
	Low in sodium	≤120 mg/100 g or 100 mL
PAHO-NPM [41]	Excessive in sodium	≥1 mg/kcal
Chile-NPM [42]	High in sodium	Solids: >400 mg/100 g Liquids: >100 mg/100 mL

**Table 2:** Foods included in the study and foods with sodium/salt content displayed.

Food Groups	No Foods	% of Total Foods	No Foods with Sodium/Salt Content	% Foods with Sodium/Salt Content ^1^
Total	3897	100	3634	93.3 ^2^
G1—Bread and bread-like cereal derivatives	148	3.8	146	98.6
G2—Canned vegetables	112	2.9	104	92.9
G3—Cereal sweet derivatives	363	9.3	356	98.1
G4—Cheese	182	4.7	167	91.8
G5—Dairies and substitutes	474	12.2	465	98.1
G6—Fats	64	1.6	58	90.6
G7—Fish/seafood—canned, processed and derivatives	273	7.0	255	93.4
G8—Meat—processed and derivatives	299	7.7	281	94
G9—Non-alcoholic drinks	249	6.4	246	98.8
G10—One type of ingredient	403	10.3	285	70.7
G11—Other processed and plant based derivatives	131	3.4	129	98.5
G12—Pasta	140	3.6	136	97.1
G13—Precooked and ready-to-eat food	224	5.7	224	100
G14—Sauces	88	2.3	75	85.2
G15—Snacks	279	7.2	276	98.9
G16—Sweets	468	12.0	431	92.1

^1^ Calculated as: No foods with sodium/salt content within each group × 100/Total No foods within each group. ^2^ Calculated as: Total No foods with sodium/salt content × 100/Total No foods surveyed.

**Table 3:** Sodium content by food group.

Food Groups	No Foods	Mean Sodium (mg/100 g)	SD (mg/100 g)	Sodium Percentiles (mg/100 g)				
				Min	25th	50th (Median)	75th	Max
Total	3616	327.8	431.9	0	32	172	520	5200
G1	144	442.3	236.3	4	339	440	560	1240
G2	104	239.5	119.5	0	169	256	345	480
G3	355	276.8	200.7	0	152	252	356	1120
G4	165	606.2	328.3	32	400	560	748	2100
G5	465	53.2	35.1	4	40	48	60	520
G6	58	186.1	244.6	0	32	52	295	1000
G7	255	692	786.6	40	400	560	600	5200
G8	280	999.2	414.6	400	720	840	1230	3480
G9	246	13.8	42.1	0	0	4	12	400
G10	276	22.7	33.1	0	4	12	30.5	212
G11	129	324.3	284.4	0	140	320	400	2000
G12	136	22.9	22.9	0	12	12	27	120
G13	223	408.1	219.3	20	248	392	520	1440
G14	75	691.8	320.8	44	480	600	816	1960
G15	274	691.1	360	0	440	680	920	2500
G16	431	45.9	68.5	0	0	28	64	576

SD: Standard deviation.

**Table 4:** Agreement between the PAHO-NPM [39] and the Chile-NPM [40].

Food Groups	κ (Confidence Interval)	Disagreement Probability ^1^	Agreement ^2^
Total	0.67 (0.66–0.68)	0.18	substantial
G1	0.67 (0.60–0.72)	0.15	substantial
G2	0.01 (−0.12–0.14)	0.87	slight
G3	0.86 (0.84–0.88)	0.04	near perfect
G4	0.41 (0.33–0.50)	0.19	moderate
G5	0.02 (−0.04–0.08)	0.31	slight
G6	0.88 (0.83–0.91)	0.03	near perfect
G7	0.18 (0.10–0.26)	0.27	slight
G8	-	0.01	near perfect *
G9	0.17 (0.09–0.25)	0.13	slight
G10	-	0.11	substantial *
G11	0.33 (0.22–0.43)	0.37	fair
G12	-	0	perfect *
G13	0.11 (0.02–0.19)	0.52	slight
G14	0.19 (0.04–0.34)	0.2	slight
G15	0.81 (0.78–0.83)	0.08	near perfect
G16	0.44 (0.39–0.49)	0.01	moderate

^1^ Values: 0 to 1. ^2^ Agreement was assessed using the κ statistic as follows: 0.01–0.20 ‘slight’; 0.21–0.40 ‘fair’; 0.41–0.60 ‘moderate’; 0.61–0.80 ‘substantial’; 0.81–0.99 ‘near perfect’. * Agreement was assessed using the disagreement probability: >0.1 ‘substantial’; <0.1 ‘near perfect’; 0 ‘perfect’.

**Table 5:** Comparison of the sodium content (mg/100 g food) by group and year.

Food Groups	Year												
	2014–16				2017–19				2020–21				*p* Value
	*n*	25th	50th (Median)	75th	*n*	25th	50th (Median)	75th	*n*	25th	50th (Median)	75th	
Total	414	90	242 ^a^	380	2455	20	100 ^a^	520	1339	52	276 ^b^	560	<0.001 *
G1	--	ND	ND	ND	67	140	400	600	78	400	480	560	0.881
G2	46	175	236	296	76	122	268	354	--	ND	ND	ND	0.265
G3	146	185.5	254	347.5	220	172	260	380	135	100	240	332	0.742
G4	--	ND	ND	ND	158	400	560 ^a^	757	79	392	600 ^b^	760	0.002 *
G5	--	ND	ND	ND	276	40	40 ^a^	60	218	40	48 ^b^	60	0.005 *
G8	--	ND	ND	ND	250	760	840 ^a^	1110	89	720	920 ^b^	1400	0.003 *
G14	--	ND	ND	ND	47	480	600 ^a^	816	32	540	680 ^b^	808	0.005 *
G15	34	600	700	900	123	306	640	980	151	520	680	840	0.071
G16	--	ND	ND	ND	345	0	28 ^a^	64	86	16	28 ^b^	51	0.005 *

* Statistically significant differences according to *p* < 0.05 by using Kruskal-Wallis test. Different lower case letters on the same line indicate significant differences in median sodium content between years. *n*: No of foods. 25th, 50th 75th: percentiles. ND: Not Determined (<30 foods or <3 brands).

## Data Availability

Not applicable.

## Supplementary Materials

The following are available online at https://www.mdpi.com/article/10.3390/nu13103410/s1, Figure S1: Sodium content of all foods in the database, Table S1: Description of the items included in the food groups, Table S2: Sodium content by specific food type, Table S3: Sodium content per serving for the precooked and ready-to eat food group, Table S4: Foods in conformity with the nutrition claims regulated by the European Regulation No 1924/2006 and Codex Alimentarius or exceeding the NPMs thresholds for sodium, by group, Table S5: Sodium content in different studies by group.
