# Supplementary material for: Sodium Content of Foods Sold in the Spanish Market. Results from the BADALI Project
Source: Nutrients. 2021 Sep 27;13(10):3410. doi: 10.3390/nu13103410 (PMC8539754; doi:10.3390/nu13103410)
Supplement: Supplementary file 1 [file nutrients-13-03410-s001.zip › nutrients-1360452-supplementary.pdf]

Supplementary Materials

Table S1. Description of the items included in the food groups.

	Food Group	Foods
**G1**	Bread and bread-like cereal derivatives	Bread, breadcrumbs, toasts, croutons, bread slices and sticks, cakes (oatmeal, rice, corn), cereal tortillas, pizza dough, precooked bread, sliced bread, hamburger and hot dog buns
**G2**	Canned vegetables	Canned vegetable and legumes
**G3**	Cereal sweet derivatives	Sweet and salty biscuits, breakfast cereals, cereal bars, pastries and pastry mix
**G4**	Cheese	All kinds of cheese, including fresh, semi-cured, cured, cottage, whipped, spreads and creams
**G5**	Dairies and substitutes	All kinds of milk including evaporated and powdered, flavoured milk shakes, coffees with milk, fermented milk, dairy desserts (curd, panna cotta, mousse, custard, rice pudding), vegetables drinks (oats, soy, wheat, spelt, rice, coconut, almond, tigernuts, nuts); vegetable alternative yogurts and desserts
**G6**	Fats	Butter, margarine, lard, cooking or whipping cream, whipped cream, vegetable cream
**G7**	Fish/seafood – canned, processed and derivatives	Canned fish and seafood (clam, anchovy, eel, anguriña, herring, tuna, cod, cockle, bonito, mackerel, crab, squid, prawn, mussel, panga, octopus, salmon, sardine, cuttlefish, scallop); broths for paella (prepared for fish or seafood paella), liver, roe and fish derivatives (surimi and spreads)
**G8**	Meat - processed and derivatives	Sausages, cold meat, luncheon meat, other processed meat, pate and spreads
**G9**	Non-alcoholic drinks	Fruit and vegetable smoothies, juices, nectars and beverages; non-alcoholic sangria, soft drinks, tonic water, soda water, non-alcoholic beer
**G10**	One-type of ingredient	Cereal cakes, cereal flakes, nuts, dried fruit, dried legumes, processed vegetables, fish and seafood (frozen and fresh), grain cereals, seeds, flour, bran, gluten, germ
**G11**	Other processed and plant based derivatives	Processed foods from plant origin with added ingredients, creams, gazpacho, spreads, hummus, tomato-based sauces, tofu, seitan
**G12**	Pasta	Pasta made of cereals, cereals with vegetables, legumes; semolina, bulgur, couscous
**G13**	Precooked and ready-to-eat food [1]	Ready-to-eat salads, meatloaf, cooked rice, chicken wings, onion rings; battered meat, vegetables, fish and squid; filled and ready-to-eat pasta dishes; churros, couscous, croquettes, falafel, nuggets, fingers, cooked vegetables, spring rolls, paellas, pizzas; vegetable alternative to processed meat (sausages, burgers, cold meat, meatballs)

**G14**	Sauces	Bechamel, mayonnaise, ketchup, barbecue, hot, mustard, allioli, bittersweet, soy, pepper, andalouse, burger, caesar, chives, chimichurri, cocktail, curry, boletus, carbonara, pedro ximénez, cheese, yogurt, kebab, pesto, vinaigrette, spicy
**G15**	Snacks	Popcorn, chips (potato, legumes), corn snacks, wheat snacks (sticks), nachos, pork fried crusts, fried vegetables; snacks elaborated with potatos, vegetables or legumes; salty nuts
**G16**	Sweets	Fruits and vegetable jams, honey, condensed milk, chocolates, chocolate bars, cocoa and derivatives, mix for chocolate products, chocolate snacks, turrón, caramelised nuts and vegetables, fruits in syrup, quince, sweets, fruit spreads

[1] Also included here those processed foods ready to eat with minimum cooking not included in other groups (according to the instructions in the pack). Foods in this group have added salt, fat and/or additives.

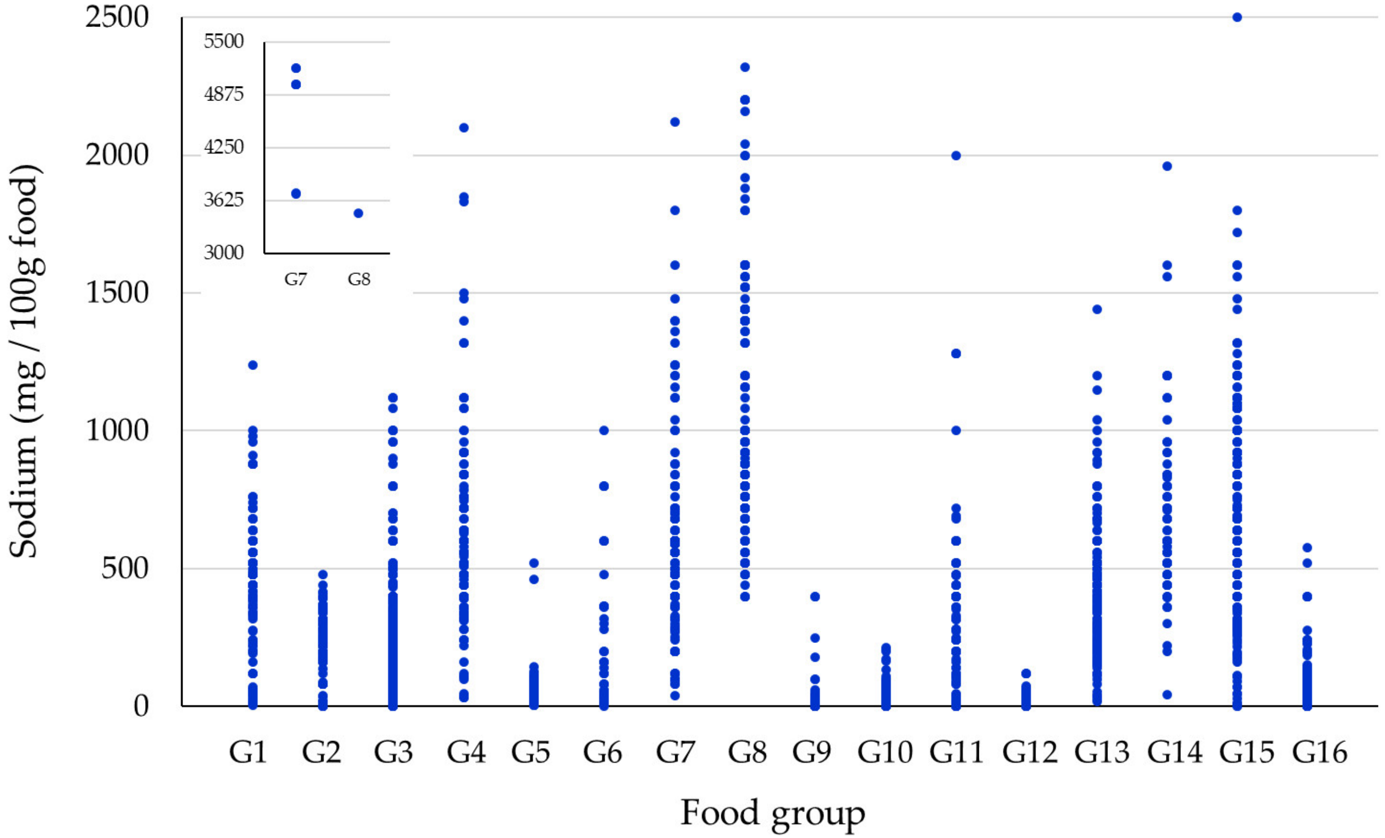

Figure S1.- Sodium content of all foods in the database

Table S2.- Sodium content by specific food type

Food group	Food type	No Foods	Median sodium (mg/100g)	25th – 75th (mg sodium/100g)	WHO benchmarks [XX]	p [1]
G1 - Bread	Bread	51	440	400 - 520	300 - 330	0.26
	Cakes and toasts	84	440	184 - 570		
G2 – Canned vegetables and legumes [2]	--	104	256	169 – 345	50	--
G3 – Cereal sweet derivatives	Bakery – pastries	36	230 [a,b]	144 - 325	120 - 205	< 0.001*
	Biscuits (sweet)	208	280 [b]	200 - 392	265	
	Breakfast cereals	82	182 [a,c]	28 – 332	280	
	Cereal bars	29	160 [c]	124 - 216	-- [3]	
G4 - Cheese	Fresh/soft	83	440	340 - 600	[4]	< 0.001
	Mature	82	640	560 - 840		
G5 – Dairies and substitutes	Dairies	354	48	40 – 60	-- [3]	< 0.001
	Substitutes	111	40	32 - 60	-- [3]	
G7 – Fish/seafood	Canned	206	520 [5]	400 - 600	360	--
	Smoked	21	880	600 – 1.240	800	--
G11 – Other processed and plant based derivatives	Tomato-based cooking sauces	17	352	200 - 400	330	--
G13	Cereal-based	63	290	200 - 360	230 - 250	--
	Pizza	10	760	690 - 790	450	--
	Salads	12	330	254 - 402	390	--
G14 - Sauces	Emulsion-based	45	560	480 - 720	500	< 0.005
	Rest (no soy sauce)	29	800	640 - 1040	650	
G15 - Snacks	Cereals	142	720 [a]	560 – 990	500 - 520	< 0.001*
	Nuts	60	420 [b]	238 – 728	280	
	Vegetables and potatos	65	560 [c]	400 - 800	500	

[1] Comparison analysis were only performed when No of foods was at least 25/food type; [2] Data from Table 3 for comparison with WHO benchmarks; [3] No benchmarks defined by WHO [25]; [4] Dissimilar subcategories with the WHO benchamarks; [5] When anchovies were not considered, as in WHO classification, median = 500 mg/100g. * P values when comparing all the food types, while different lower case letters indicate significant differences by pair of food types.

Table S3.- Sodium content per serving for the precooked and ready-to eat food group

		Sodium (mg)/serving (n=141)	% IR
	**Mean**	591.1	29.6
	**SD**	374.3	18.7
**Percentiles**	**Min**	50	2.5
	**25th**	304	15.2
	**50th (Median)**	507.5	25.4
	**75th**	800	40
	**Max**	2296	114.8

SD: Standard deviation, % IR: Percentage of recommended intake for adults (2 g sodium/day) [10]

Table S4.- Foods in conformity with the nutritional claims regulated by the European Regulation No 1924/2006 [39] and Codex Alimentarius [40] or exceeding the NPMs thresholds for sodium, by group.

Food Groups	Sodium-free (%) [1]	Very low sodium (%) [1]	Low sodium (%) [1]	High sodium	
				PAHO - NPM (%) [1]	Chile - NPM (%) [1]
Total	460 (12.7) [2]	1171 (32.4) [2]	1744 (48.2) [2]	1705 (47.2) [2]	1153 (31.9) [2]
G1	1 (0.2)	10 (0.9)	21 (1.2)	104 (6.1)	86 (7.5)
G2	4 (0.9)	10 (0.9)	22 (1.3)	94 (5.5)	4 (0.3)
G3	9 (2)	34 (2.9)	77 (4.4)	55 (3.2)	54 (4.7)
G4	0 (0)	3 (0.3)	8 (0.5)	149 (8.7)	117 (10.1)
G5	3 (0.7)	204 (17.4)	461 (26.4)	145 (8.5)	4 (0.3)
G6	1 (0.2)	27 (2.3)	36 (2.1)	9 (0.5)	11 (1)
G7	0 (0)	1 (0.1)	12 (0.7)	241 (14.1) [3]	172 (14.9)
G8	0 (0)	0 (0)	0 (0)	279 (16.3)	277 (24)
G9	145 (31.5)	238 (20.4)	242 (13.9)	37 (2.2)	4 (0.3)
G10	109 (23.7)	238 (20.4)	270 (15.5)	31 (1.8)	0 (0)
G11	9 (2)	19 (1.6)	31 (1.8)	100 (5.9)	52 (4.5)
G12	20 (4.3)	105 (9)	136 (7.8)	0 (0)	0 (0)
G13	0 (0)	5 (0.4)	12 (0.7)	206 (12.1)	90 (7.8)
G14	0 (0)	0 (0)	1 (0.1)	60 (3.5)	69 (6)
G15	3 (0.7)	6 (0.5)	14 (0.8)	189 (11.1)	210 (18.2)
G16	156 (33.9)	271 (23.2)	401 (23)	6 (0.4)	3 (0.3)

[1] Calculated as No foods complying with the criteria per group x 100 / Total No foods complying with the criteria. [2] Calculated as Total No foods complying with the criteria x 100 / Total No foods surveyed. [3] n = 252: the criteria could not be applied to three foods because the kcal content could not be calculated

Table S5. Sodium content in different studies by group [1].

Food Group	Country	No Foods	Sodium (mg/100g)		Comments / Reference
			Mean	Median	
**Bakery and cakes**	**BADALI** [2]	355	276.8	252	Cereal sweet products, including bakery
	Argentina	101	299.5	250	[54]
	Costa Rica	58	341	344	Data for cakes / [28]
	Latin America	1443	383	328	Data for cakes / [55]
	USA	520	447.6	--	[29]
**Bread**	**BADALI** [2]	144	442.3	440	
	Argentina	110	442.7	443	[54]
	Costa Rica	87	291-448	291-480	Data for bread and wholemeal bread / [28]
	Italy	147	600	--	[56]
	Latin America	1271	465	458	[55]
	Slovenia	126	546	--	[30]
	South Africa	174	542	476	[57]
	Spain	1137	832	--	[47]
	UK	1651	405	400	[58]
	USA	4466	455	459	[58]
**Canned vegetables**	**BADALI** [2]	104	239.5	256	Canned vegetables and legumes
	Argentina	147	251.6	196,9	[54]
	Slovenia	330	484	--	[30]
	South Africa	895	288	108	Vegetables in general / [57]
	USA	155	228.9	--	Vegetables in general / [29]
**Cheese**	**BADALI** [2]	165	606.2	560	
	Argentina	316	628.5	583,3	[54]
	Latin America	1911	739	643	[55]
	Slovenia	292	524	--	[30]
	South Africa	240	654	554	[57]
	**BADALI** [2]	255	692	560	
	Argentina	123	655.9	411,7	[54]

**Fish and fish products**	Slovenia	155	659	--	Canned fish and seafood; 7 samples without salt were not included in the analysis / [30]
	South Africa	284	384	328	[57]
**Meat**	**BADALI** [2]	280	999.2	840	
	Argentina	253	956.5	843,8	[54]
	Latin America	2071	928	870	[55]
	Slovenia	362	984	--	[30]
	South Africa	545	850	734	[57]
	USA	396	1014.8	--	[29]
**Ready meals**	**BADALI** [2]	223	408.1	392	Precooked and ready-to-eat food
	Slovenia	206	510	--	[30]
	South Africa	156	422	382	[57]
**Sauces**	**BADALI** [2]	75	691.8	600	
	Argentina	214	1247.3	850	Sauces and spreads / [54]
	India	271	2217	--	Sauces and spread / [31]
	Malaysia	117	3164	--	[59]
	Slovenia	273	1131	--	[30]
	South Africa	1059	1981	673	Sauces and spreads / [57]
	UK	918	732	360	[32]
	USA	274	613.4	--	[29]
**Snacks**	**BADALI** [2]	274	691.1	680	
	Argentina	240	937.1	683,7	[54]
	India	200	666	--	[31]
	Latin America	2235	724	625	[55]
	Slovenia	206	787	--	[30]
	USA	150	823.4	--	[29]

[1] Only food groups which typically contain added salt and studies of at least 50 foods per group are included. [2] Present work
